# Endoscopic sleeve gastroplasty (ESG): indications and results—a systematic review

**DOI:** 10.1007/s13304-025-02097-1

**Published:** 2025-01-28

**Authors:** Christine Karolina Stier, Patrick Téoule, Barham K. Abu Dayyeh

**Affiliations:** 1 Department of Surgery, Universitätsmedizin Mannheim, Medical Faculty Mannheim University of Heidelberg, Theodor-Kutzer-Ufer 1-3, 68167 Mannheim, Germany; 2 Department of General and Visceral Surgery, Ulm University Hospital, Ulm, Germany; 3 Division of Gastroenterology and Hepatology, Mayo Clinic, Rochester, MN USA; 4 Digestive Health Institute, University of California, Irvine, California USA

**Keywords:** Obesity, Bariatric endoscopy, Endoscopic sleeve gastroplasty, Primary weight loss procedure, Excess weight loss, Long-term results, Safety

## Abstract

Obesity is a major global health problem and at the same time a financial burden for social security systems. For a long time, conventional lifestyle interventions have tried unsuccessfully to find a solution. It has been proven that only interventions that ultimately address the central control centers of hunger, appetite and satiety will lead to sustained weight loss. As a result, metabolic and bariatric surgery (MBS) has become the gold standard in the treatment of obesity and has been shown to be effective and safe in both the short and long term. Processed via the gut–brain axis, MBS not only leads to weight loss, but also—and, in addition, independently through the modification of the intestinal tract in bypass surgery—to a significant remission rate of type 2 diabetes mellitus, the typical co-morbidity of obesity. However, MBS is not suitable for all patients. Some patients are ineligible due to a high-risk profile or do not wish to undergo surgery, whilst others do not meet the criteria for MBS but still suffer from obesity. This treatment gap has been a driving force behind the development of endoscopic solutions such as endoscopic sleeve gastroplasty (ESG). ESG offers a less invasive, endoluminal and anatomy-sparing alternative that reduces gastric volume by suturing tissue folds along the greater curvature. Such a reduction in gastric volume, which is also one principle of action of MBS, can induce earlier satiety and lead to weight loss. The evidence behind this procedure, in particular its efficacy and safety, should be objectified through this review.

## Background

Obesity rates have been rising for decades and are now skyrocketing. The World Health Organisation (WHO) has long warned of its health consequences and in 2006 called on its European members to recognise obesity as a chronic disease so that patients from all backgrounds can receive regular treatment. However, regional, cultural, and socio-economic factors contribute to differences in prevalence and incidence around the world [1], which is currently estimated at around 40% of the population and is predicted to reach 51% of the US population by 2030 [2]. These alarming estimates call for a strategic treatment plan. Viewed from this perspective, surgical treatment remains the most sustainable and successful evidence-based therapeutic approach, including the management of associated comorbidities. Although it is safe, it is the most invasive therapeutic option available, but one that promotes sustained weight loss and a reliable reduction in all-cause mortality rates [3]. Ultimately, the analysis of the astonishing therapeutic success of MBS led to the development of both the new but already ubiquitously promoted incretin drugs and the development of endoscopic weight loss procedures. In a way, they mimic its mode of action. As a result, there are now three effective interventional treatments for obesity—drugs, endoscopy, and surgery—each with its individual indication, long-term effect, and success.

Until recently, MBS was indicated from a BMI of 40 kg/m^2^ (obesity class III) or 35 kg/m^2^ (obesity class II) with comorbidities, while both medical and endoscopic procedures were recommended from a BMI of 30 (obesity class I) kg/m^2^ or 27 with comorbidities. Each of these interventions should be preceded by 6 months of conservative therapy, which must have been unsuccessful as a prerequisite. To reflect the current situation, the new guidelines for MBS have adjusted the BMI limits for surgery downwards by five points and it has also lately been proposed for mild obesity associated with refractory metabolic disease [4]. However, only 1–2% of eligible patients undergo surgery. This leaves a large treatment gap between the needs of patients suffering from obesity and what we can offer in terms of medical, endoluminal and surgical interventions [5]. It is this unmet need that has driven the development of endoscopic solutions for obesity, particularly where MBS is not feasible, desired or even warranted.

**Fig. 1:**
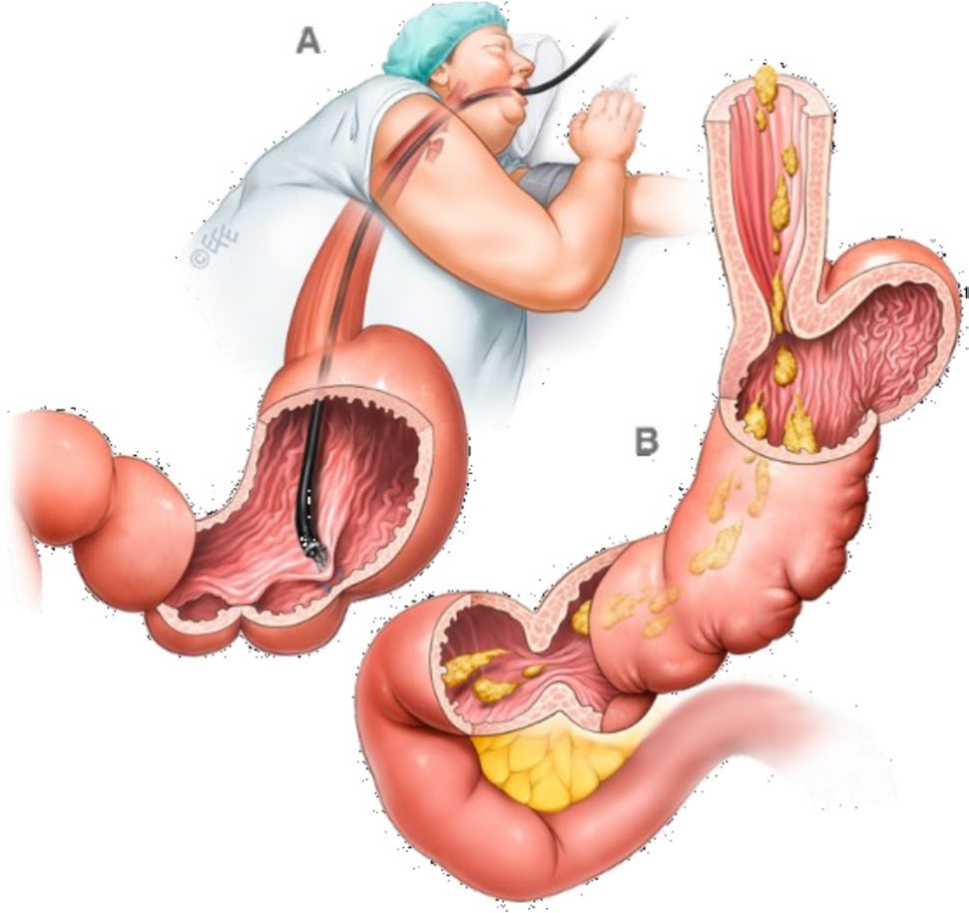
Endoscopic sleeve gastroplasty (ESG), courtesy of IFSO

Endoluminal gastroplasty is one such solution that has gained global acceptance in recent years and can be performed using a variety of suturing devices. The best known is endoscopic sleeve gastroplasty (ESG), in which sutures are placed in specific patterns along the great curvature of the stomach to significantly reduce endoluminal gastric volume by creating apposition of the anterior against the posterior wall of the stomach. There are several advantages to endoscopic bariatric procedures. These include particularly anatomical preservation and an improved risk profile. To effectively combat obesity, these benefits also allow for a personalised gradation of procedures offered. In its current clinically adopted and approved form [6], ESG utilises the Overstitch™ platform (former Apollo EndoSurgery, Austin, TX, USA, now Boston Scientific, Marlborough, MA, USA), which allows for full-thickness endoscopic suturing and simultaneously for running sutures [7, 8]. Suturing begins at the junction of the gastric body and the antrum and progresses upwards towards the fundus, which is usually largely preserved, thus creating a small pouch to allow for fundal accommodation of food. Prolonged gastric accommodation is one significant part of creating satiation. Early satiety is facilitated by the tubular gastric body [9]. These physiological principles of facilitating a feeling of fullness and satiety, achieved by endoluminal modulation of anatomy, are consistent, making the effect of the procedure reproducible and the procedure itself clinically mature [10] (Fig. 1).

## Review incorporating the comprehensive meta-analysis-based IFSO Bariatric Endoscopy Committee Position Statement on endoscopic sleeve gastroplasty for obesity treatment [11]

The evidence base for the efficacy and safety of ESG continues to grow and has been the subject of more than 200 publications [11]. The currently available evidence has been supplemented and confirmed by an open-label, multicentre, randomised trial with 24-month follow-up, the results of which were presented in 2022 [12]. The procedure is currently used worldwide, with more than 40,000 clinical procedures performed to date.

Recently, the Endoscopy Committee of the International Federation of the Surgery of Obesity and Related Diseases (IFSO) released a comprehensive systematic review of the evidence for ESG to support the consensus statements on the procedure, which serves as the basis for this review, which is summarised here.

## Methodology

This review is based entirely on the previously published position statement of the Bariatric Endoscopy Committee of the International Federation for the Surgery of Obesity and Related Diseases (IFSO), which is a comprehensive meta-analysis on the subject. (IFSO Bariatric Endoscopy Committee Evidence-Based Review and Position Statement on Endoscopic Sleeve Gastroplasty for Obesity Management by Barham K Abu Dayyeh, Christine Stier, Aayed Alqahtani, Reem Sharaiha, Mohit Bandhari, Silvana Perretta, Sigh Pichamol Jirapinyo, Gerhard Prager and Ricardo V Cohen). The background literature search, conducted by two independent researchers from the Mayo Clinic on behalf of the IFSO Endoscopy Committee, covered the period from 1 January 2013, the year the topic was first published, to 1 October 2022 (last search update), searching MEDLINE (Pubmed), EMBASE and grey literature. The original paper, on which this review is fully based, provides an excellent detailed description of the technical basis and state-of-the-art methodology used during the meta-analysis review, including the assessment of risk of bias using the Joanna Briggs Institute Critical Appraisal checklist for case series, the New-Castle Ottawa scale for cohort studies, the JADAD score and a modified Cochrane Collaboration Risk of Bias tool (https://www.ncbi.nlm.nih.gov/books/NBK132494/bin/appf-fm1.pdf) [11]. Preferred Reporting Items for Systematic Reviews and Meta-Analyses (PRISMA) and Cochrane guidelines were followed. The source meta-analysis is based on 75 references listed in the original article, available at Obesity surgery (https.//doi.org/10.1007/s11695-024-07510-z).

The IFSO Endoscopy Committee’s review primarily examined weight loss outcomes and safety data of ESG performed with the Overstitch™ platform (former Apollo EndoSurgery, Austin, TX, USA; now Boston Scientific, Marlborough, MA, USA).

Inclusion criteria required ESG to be performed using the Overstitch™ device but did not mandate specific suture pattern. Articles had to be in full text in any language. The sample size had to comprise at least ten individuals, but could have been conducted as case series, cohort studies, case–control studies or, at best, randomised trials.

**Fig. 2:**
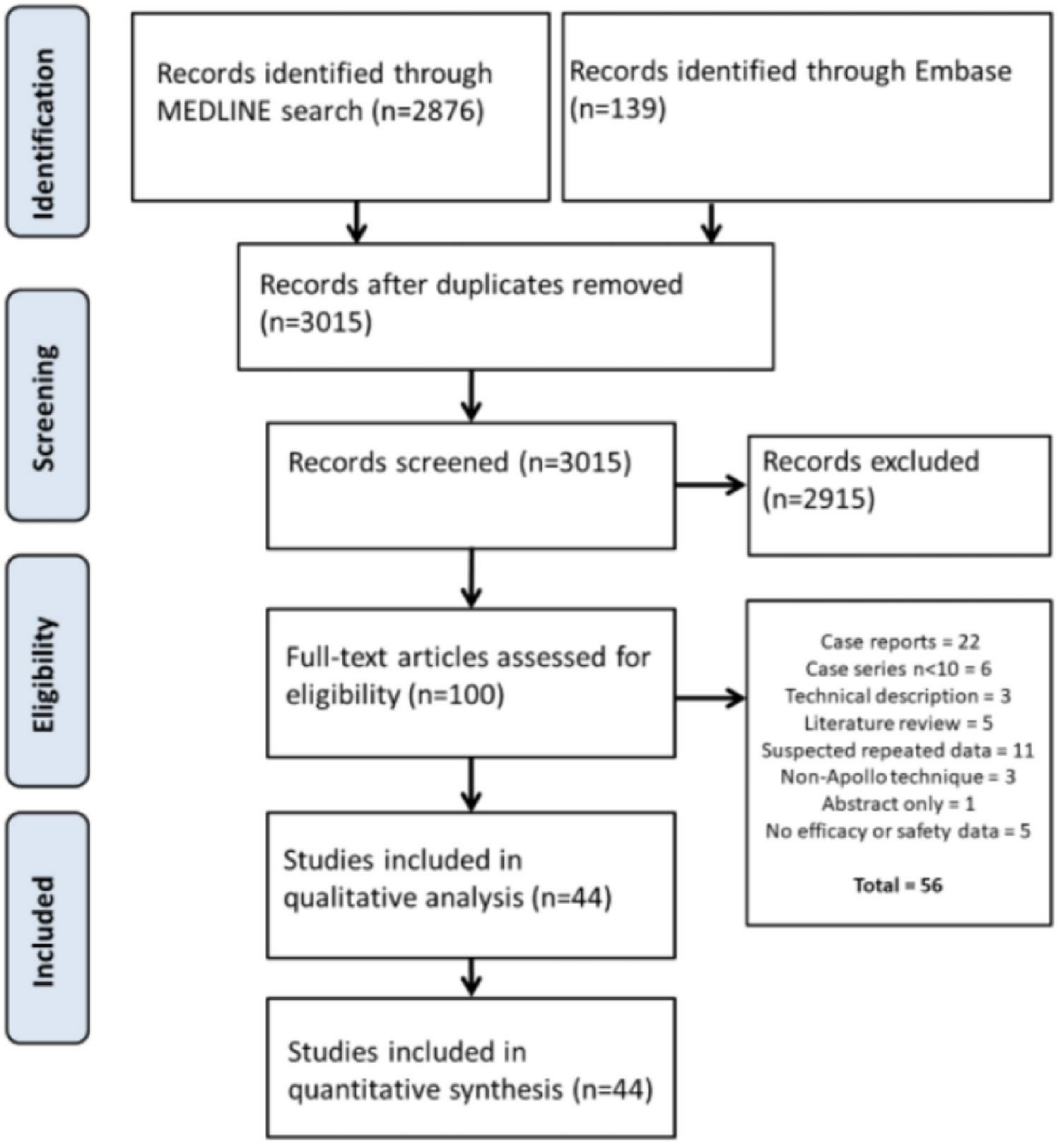
PRISMA flowchart for the literature screening and inclusion/exclusion process for the overall outcomes of ESG (non-comparative analysis)

From the initial 3015 retrieved records, a total of 100 articles were selected for full-text assessment. Lastly 44 articles (29 case series, 14 cohort studies and 1 randomised controlled trial (RCT) were included in the quantitative synthesis respectively qualitative analysis. The PRISMA diagram illustrates the flow of information through the stages of the source systematic review and meta-analysis, providing the most up-to-date evidence (Fig. 2) [11].

Within the 14 cohort studies, 7 compared ESG with laparoscopic sleeve gastrectomy, 1 compared ESG with lifestyle intervention alone, 1 compared ESG with ESG plus adjuvant anti-obesity medication, 2 compared ESG with intragastric balloons, and 3 examined different stitching patterns within ESG cohorts.

Corresponding ESG results were extracted from comparative studies to be included in the non-comparative meta-analysis to describe outcomes at specific time points (6, 12, 18, 24, 36 and 60 months). Endpoints of statistical evaluation were the already above-mentioned efficacy and the procedure’s safety profile.

In addition, the pooled evidence was graded into four different categories: VERY LOW, LOW, MODERATE and HIGH, using the Grading of Recommendations, Assessment, Development and Evaluations (GRADE) approach.

## Results

The non-comparative analysis of the 44 articles included 49,848 patients, whereas 15,714 patients underwent ESG with 34,134 controls, including laparoscopic sleeve gastrectomy and adjustable gastric banding respectively intragastric balloons. The majority of patients were female, with 11,449 women (83.2%) and 2,304 men (16.8%) included in 42 articles (*n* = 13,753). The average baseline age and BMI were 44.24 years (SE 1.405, 95% CI 41.48–46.99; 41 articles, *n* = 13,562) and 37.56 (SE 0.45, 95% CI 36.66–38.46; 42 articles, *n* = 13,876), respectively.

*Safety*. The meta-analyses regarding the safety of ESG, revealed within 40 articles, reporting the occurrence of serious adverse events at a pooled rate of 1.25% (*n* = 194 events; amongst 15,398 ESG procedures). Within the currently available evidence, these results underline the safety of ESG with regard to short- and long-term results. Common adverse events associated with ESG include nausea, vomiting and abdominal discomfort. More serious adverse events have been associated with bleeding and perigastric fluid collection, which may require re-intervention. These were not distinguished in this particular source meta-analysis but are detailed in the underlying literature. [11].

**Fig. 3:**
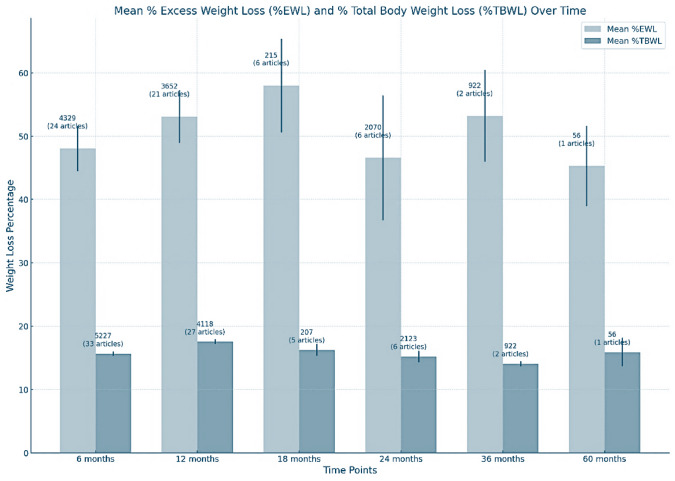
Mean percentage of excess weight loss (%EWL) and total body weight loss (%TBWL) at specific time points following endoscopic sleeve gastroplasty (ESG). For context, the number of individuals and articles at each time point are included above the bars

*Weight loss*. The weight loss outcome parameters depending on the time points after ESG are summarised in Table 1. After 6 months, the %- excess weight loss (EWL) was nearly 48% (standard error 3.59, 95% CI 40.98–55.09, 24 articles, 4329 patients), respectively, the %- total body weight loss (TBWL) was about 16% (standard error 0.35, 95% CI 14.95—16.36, 33 articles, 5227 patients). During the maximum reported follow-up period of 5 years, the %EWL approximately reached 45 (standard deviation 47.32, 1 article, 56 patients) and 16% of TBWL (standard deviation 16.79, 1 article, 56 patients). Within the currently available evidence, these results underline the efficacy of ESG with regard to short- and medium-term outcomes of weight loss (Fig. 3).

**Table 1:** Meta-analyses of weight loss parameters after ESG [11]

Follow-up after ESG (months)	Mean %EWL	Mean % TBWL
6	48.04	15.66
12	53.09	17.56
18	57.98	16.25
24	46.57	15.2
36	53.18	14.07
60	45.3	15.9

*ESG* endoscopic sleeve gastrectomy, *EWL* excess weight loss, *TBWL* total body weight loss

*Meta-analysis*. Regarding the comparison of ESG and lifestyle intervention, two studies were eligible for quantitative synthesis and qualitative analysis, respectively. The first one, a case-matched cohort study (1 ESG: 2–3 controls), was published in 2020 by Cheskin et al. [13] and included patients with obesity grad I or higher. Authors compared ESG combined with low-intensity diet and lifestyle therapy (LIDLT) against high-intensity diet and lifestyle therapy (HIDLT). Costs were covered by the patients themselves, with a total amount of 16,000 $ for ESG and 3200 $ HIDLT. In total, 386 patients (105 ESG, 281 controls) were enrolled with comparable baseline characteristics, with the final follow-up at 1 year. The second one, a multi-centre, US FDA-regulated, open-label, RCT, was published in 2022 by Abu Dayyeh et al. and only included patients with obesity grade I and II [12]. The authors compared ESG plus lifestyle interventions with lifestyle interventions alone (MERIT Trial). Patients were randomly assigned to ESG or control groups in a 1:1.5 ratio. In total, 209 patients (85 ESG, 124 controls) were included, also with similar baseline characteristics. Within the MERIT Trial, the primary endpoints were %EWL and %TBWL at 1 year, with an extended follow-up at 24 months for the intervention group and a 12-month follow-up for patients in the control group crossing over to the intervention group. As per the Cochrane Handbook, data from different study designs should not be combined when only a few eligible studies are available. Consequently, due to the fact that the two studies differ in design (case-matched cohort study vs. RCT) and population (non-specified obesity vs. grade I and II), the data were analysed separately. The case-matched cohort study by Cheskin et al. [13] revealed after 12 months, a mean difference in %TBWL of 6.3 [95% CI 3.12–9.48] after ESG with LIDLT (mean:20.6, SD:8.3; *n* = 43) compared to the control group with HIDLT (mean:14.3, SD:10.2; *n* = 101). The RCT by Abu Dayyeh et al. [12] showed, after 12 months, a mean difference in %EWL of 46.00 [95% CI 38.05–53.95] and a mean difference in %TBWL of 13.10 [95% CI 11.08–15.12] after ESG combined with lifestyle interventions (%EWL—mean: 49.2, SD:32; and %TBWL—mean:13.6, SD:8; *n* = 77) compared to lifestyle interventions alone (%EWL—mean:3.2, SD:18.6; and %TBWL—mean:0.5, SD:5; *n* = 110). Regarding the occurrence of serious adverse events, the rate was 2% without mortality or need for intensive care or surgery intervention.

It has to be taken into account that the review included many observational studies, despite being of very low quality of evidence, reported consistently positive outcomes across various global settings, demonstrating ESG’s reproducibility and generalizability. A single RCT was included in the meta-analysis and provided moderate quality of evidence, further validating ESG’s efficacy and safety. This combination of evidence supports the IFSO’s 2023 Delphi Consensus statement on ESG’s role in obesity care.

*Indications*. The above-mentioned IFSO statements have been developed for the purpose of defining the specific indications for the ESG in the context of obesity treatment. In 2023, all available evidence was presented to a multidisciplinary committee of experts convened on behalf of the IFSO. Using a Delphi method, recommendations on the usefulness of ESG for the management of obesity were agreed and endorsed by the expert conference. Based on the presented data, the IFSO consensus supports ESG as an effective intervention over lifestyle alone in patients with obesity grades I and II and those with grade III who are unwilling or ineligible for conventional MBS (Table 2), as this minimally invasive procedure achieves significant short- and medium-term %EWL and %TBWL combined with a low rate of serious adverse events, and therefore a reasonable risk profile.

**Table 2:** IFSO statements from the 2023 Consensus Conference on ESG indications [14]

• ESG combined with lifestyle intervention is preferable to lifestyle interventions alone, for the management of adults with class I obesity
• ESG combined with lifestyle intervention is preferable to lifestyle interventions alone, for the management of adults with class II obesity
• ESG combined with lifestyle is an acceptable management option for adults with class III obesity who either do not qualify (given medical or psychological comorbidities) or do not wish to pursue MBS
• ESG combined with lifestyle intervention is preferable to lifestyle interventions alone, for the management of adolescents with class II obesity

*ESG* endoscopic sleeve gastrectomy

Notably, the expert committee, which included as well paediatric endocrinologists, also endorsed for the first time the use of the procedure to treat adolescents with class II obesity who have not responded to conservative therapy [14].

In general, the endoscopic procedure should be complemented by a multidisciplinary obesity programme. These statements by the IFSO Consensus Committee (Table 2) were confirmed by the consensus statements of the UK National Institute for Health and Care Excellence (NICE) guidelines, which also made almost identical recommendations on the benefits of ESG in the adult population, based on their own analysis of the evidence (Table 3).

**Table 3:** NICE guideline statements on ESG indications [15]

The committee considered that this procedure may particularly benefit people:
• with class 3 obesity for whom invasive bariatric surgery would be considered high risk
• who decline bariatric surgery because of the associated risks and complications
• who have class 1 or class 2 obesity, for whom the procedure may prevent progression of obesity and associated comorbidities

The (NICE) committee noted that evidence included people with obesity (a body mass index [BMI] over 30 kg/m2) for whom non-surgical weight loss treatments had not worked, and people with class 3 obesity for whom invasive bariatric surgery would be considered high risk

*NICE* National Institute for Health and Care Excellence, *ESG* endoscopic sleeve gastrectomy

These are the two most important consensus statements available that the ESG recommends for a clearly defined range of indications.

## Future direction

Based on the integrated comprehensive systematic review and meta-analysis of the IFSO Bariatric Endoscopy Committee, only ESG with the Overstitch™ platform (former Apollo EndoSurgery, Austin, TX, USA, now Boston Scientific, Marlborough, MA, USA) is included and presented. This is driven by the maturity of the technology and the regulatory approvals already achieved.

Other endoscopic gastric remodelling techniques, such as Primary Obesity Surgery Endoluminal 2.0 (USGI Medical, San Clemente, CA), Endomina™ Gastric Plication (Endo Tools, Gosselies, Belgium), and the Endozip™ automated suturing device (Caesarea, Israel), are at various stages of clinical trials and evidence level, showing similar safety and efficacy profiles. As new clinical evidence emerges for these procedures, the IFSO Bariatric Endoscopy Committee will take them into account during the future revision of their statement.

Recent advancements in obesity pharmacotherapies now offer effective options for certain patients suffering from obesity. The comparative effectiveness of ESG versus or in combination with these pharmacotherapies is an ongoing area of research. Observational studies have highlighted the benefits of combining or sequencing ESG with obesity pharmacotherapies, particularly in improving the durability of response. The international consensus on combination therapies on the role of obesity management medications in the context of bariatric surgery—including a chapter on bariatric endoscopy—was recently published as expert guideline by the IFSO in the British Journal of Surgery [16].

In addition, an RCT comparing the effects of ESG with pharmacotherapy in adolescents, led by the IRCAD institutes in India and France, has recently begun recruiting patients.

In conclusion, however, current literature, which generally includes follow-up data for 5 years or less, is not robust enough to fully understand long-term responses of ESG. Therefore, improving the durability of response and thus long-term health outcomes are other objectives that need to be targeted in more detail. In particular, to better understand and define the use of personalised strategies, more data are needed.

## Data Availability

The original review and meta-analysis is published with Springer Nature Obesity Surgery as Position statement of the IFSO Bariatric Endoscopy Committee and includes all data of the underlying publications.

## References

[1] Worldwide trends in body-mass index (2017) underweight, overweight, and obesity from 1975 to 2016: a pooled analysis of 2416 population-based measurement studies in 128·9 million children, adolescents, and adults. Lancet 390:2627–2642 29029897 10.1016/S0140-6736(17)32129-3 PMC5735219

[2] Finkelstein EA, Khavjou OA, Thompson H et al (2012) Obesity and severe obesity forecasts through 2030. Am J Prev Med 42:563–570 22608371 10.1016/j.amepre.2011.10.026

[3] Carlsson LMS, Sjöholm K, Jacobson P et al (2020) Life expectancy after bariatric surgery in the Swedish obese subjects study. N Engl J Med 383:1535–1543 33053284 10.1056/NEJMoa2002449 PMC7580786

[4] Eisenberg D, Shikora SA, Aarts E et al (2022) 2022 American Society for Metabolic and Bariatric Surgery (ASMBS) and International Federation for the Surgery of Obesity and Metabolic Disorders (IFSO): Indications for Metabolic and Bariatric Surgery. Surg Obes Relat Dis 18:1345–1356 36280539 10.1016/j.soard.2022.08.013

[5] Ogden CL, Carroll MD, Kit BK et al (2014) Prevalence of childhood and adult obesity in the United States, 2011–2012. JAMA 311:806–814 24570244 10.1001/jama.2014.732 PMC4770258

[6] Abu Dayyeh BK, Rajan E, Gostout CJ (2013) Endoscopic sleeve gastroplasty: a potential endoscopic alternative to surgical sleeve gastrectomy for treatment of obesity. Gastrointest Endosc 78:530–535 23711556 10.1016/j.gie.2013.04.197

[7] Galvao-Neto MDP, Grecco E, Souza TFd, et al. Endoscopic sleeve gastroplasty - minimally invasive therapy for primary obesity treatment. Arquivos brasileiros de cirurgia digestiva : ABCD = Brazilian archives of digestive surgery 2016;29Suppl 1:95–97. 10.1590/0102-6720201600S10023 PMC5064280 27683786

[8] Lopez-Nava G, Galvão MP, Bautista-Castaño I et al (2015) Endoscopic Sleeve Gastroplasty: How I Do It? Obes Surg 25:1534–1538 26003549 10.1007/s11695-015-1714-7

[9] Vargas EJ, Rizk M, Gomez-Villa J, et al. Effect of endoscopic sleeve gastroplasty on gastric emptying, motility and hormones: a comparative prospective study. Gut 2022. 10.1136/gutjnl-2022-327816 PMC10102256 36241388

[10] López-Nava Breviere G, Bautista-Castaño I, Fernández-Corbelle JP et al (2016) Endoscopic sleeve gastroplasty (the Apollo method): a new approach to obesity management. Rev Esp Enferm Dig 108:201–206 26900986 10.17235/reed.2016.3988/2015

[11] Abu Dayyeh BK, Stier C on the behalf of the IFSO Endopscopy Committee. IFSO Bariatric Endoscopy Committee Evidence-Based Review and Position Statement on Endoscopic Sleeve Gastroplasty for Obesity Management. 10.1007/s11695-024-07510-z PMC11671576 39482444

[12] Abu Dayyeh BK, Bazerbachi F, Vargas EJ et al (2022) Endoscopic sleeve gastroplasty for treatment of class 1 and 2 obesity (MERIT): a prospective, multicentre, randomised trial. Lancet 400:441–451 35908555 10.1016/S0140-6736(22)01280-6

[13] Cheskin LJ, Hill C, Adam A et al (2020) Endoscopic sleeve gastroplasty versus high-intensity diet and lifestyle therapy: a case-matched study. Gastrointest Endosc 91:342-349.e1 31568769 10.1016/j.gie.2019.09.029

[14] Salminen P, Kow L, Aminian A et al (2024) IFSO consensus on definitions and clinical practice guidelines for obesity management-an international delphi study. Obes Surg 34:30–42 37999891 10.1007/s11695-023-06913-8 PMC10781804

[15] National institute for health and care excllence (NICE). Endoscopic Sleeve Gastroplasty for Obesity. Interventional procedures guidance Published: 22 February 2024

[16] Cohen RV, Busetto L, Levinson R, et al. International Consensus on the Role of Obesity Management Medications in the Context of Metabolic Bariatric Surgery. International consensus position statement on the role of obesity management medications in the context of metabolic bariatric surgery: expert guideline by the International Federation for the Surgery of Obesity and Metabolic Disorders (IFSO). Br J Surg. 2024 Nov 27;111(12):znae283. 10.1093/bjs/znae283 39612579

